# Cardiorespiratory Events in Children During Chemoembolization of Ophthalmic Artery for Retinoblastoma

**DOI:** 10.7759/cureus.16630

**Published:** 2021-07-26

**Authors:** Maria Cristina Martinez-Ávila, Ruben Carrasquilla, Antonio Oyola, Jonathan Rodriguez, Angel Castro-Dager, Rafael Almeida Pérez, Gina De la Rosa, Fernando Orozco-Gómez, Miguel Quintero-Consuegra

**Affiliations:** 1 Intensive Care Unit, Nuevo Hospital Bocagrande, Cartagena, COL; 2 Anesthesiology and Perioperative Medicine, Neurodinamia, Cartagena, COL; 3 Anesthesia and Critical Care, Neurodinamia, Cartagena, COL; 4 Pain Medicine, Neurodinamia, Cartagena, COL; 5 Haematology, Universidad Nacional, Cartagena, COL; 6 Neurological Surgery, Neurodinamia, Cartagena, COL; 7 Neuroradiology, Neurodinamia, Cartagena, COL; 8 Research and Development, Cedars-Sinai Medical Center, Los Angeles, USA

**Keywords:** retinoblastoma, cerebral angiography, chemoembolization, intraoperative complication, super selective ophthalmic artery chemoembolization

## Abstract

Retinoblastoma (RB) is the most common eye neoplasm in childhood, accounting for 2.5% to 4% of all pediatric cancers. Recent approaches to treat RB include localized administration of chemotherapy, such as super-selective ophthalmic artery chemotherapy. Although localized chemotherapy aims that minimize systemic side effects, some adverse cardio-respiratory reactions have been described as associated with this therapy. Our case report describes the cardiorespiratory severe events presented secondary to super-selective chemotherapy of the ophthalmic artery, to which we must be prepared to avoid fatal outcomes

## Introduction

Retinoblastoma was first described by Petras Pawius in 1597. The tumor currently known as ‘RB’ is the most common eye neoplasm in children. It accounts for 2.5 to 4% of all pediatric tumors, with an incidence in the USA and Europe of two to five cases per million [1]. It carries a significant hereditary component [2], with an autosomal dominant inheritance pattern associated with the RB1 gene mutation, which could lead to visual impairment in more than one family member. RB is diagnosed at an average age of 18 months. Leukocoria is the most common finding at presentation, other common symptoms are strabismus and vision loss. Neovascularization, neovascular glaucoma, hyphema, pseudohypopyon and vitreous hemorrhage may be observed in advanced cases. 

Until 1990, the treatment of RB was based on enucleation and radiotherapy, which eventually evolved into systemic chemotherapy. Adjuvant chemotherapy provides good cure rates, survival rates exceed 95-98%, as it takes care of any systemic metastasis. Side effects related to systemic chemotherapy include bone marrow suppression, alopecia, autotoxicity and nephrotoxicity

Nowadays, ophthalmologists, pediatric oncologists, radiation oncologists and neurointerventional radiologists work as a team in the treatment process. The goals of treatment have now shifted to emphasize eye salvage and vision preservation in addition to patient survival. To generate a more localized treatment to prevent systemic adverse reactions while preserving the eye, novel treatments have emerged such as the super-selective intraarterial chemotherapy of the ophthalmic artery (SOAC) [3]. Unlike systemic chemotherapy, this super-selective treatment requires the patients to be under general anesthesia. General anaesthetics have some common side effects, possible risks include anaphylaxis, accidental awareness, nerve injury, hypothermia, sore throat and laryngeal damage. Others serious complications include respiratory depression, cardiovascular collapse. Here we present two cases of patients with RB treated with SOAC and presented severe cardiovascular adverse events.

## Case presentation

Case 1

A five-year-old male patient with left congenital retinoblastoma was admitted for a second chemotherapy session of SOAC. Previous clinical history included bronchiolitis and SOAC with Melphalan without any complications. The anesthesia induction was done with sevoflurane 4% and O2, and anesthesia maintenance with sevoflurane and remifentanil, in addition to atropine 0.1 mg IV. After canalization of the ophthalmic artery, the patient presented a sudden decrease of pulmonary distention, hypoxemia (SaO2 30%), and bradycardia that soon converted into pulseless electrical activity. Code blue was activated, and cardiopulmonary resuscitation (CPR) maneuvers were executed, including 10 ucg/kg of adrenaline and cardiac massage during three minutes. The patient returned to spontaneous circulation, and the chemoembolization was changed from super selective to non-selective chemotherapy in the internal carotid artery. The patient was transferred to the pediatric ICU. With favorable evolution, he was extubated at 24 hours post-surgery and discharged without any neurological deficit three days after.

Case 2 

A 17-month-old female patient with a diagnosis of bilateral retinoblastoma was admitted for the second session of SOAC. Family history included RB in her father and aunt from her father's side of the family. The patient had received systemic chemotherapy with Melphalan and the first session of SOAC that did not present with any complication. For the second session of SOAC, the patient received anesthetic induction with inhaled propofol, and rocuronium, and general anesthesia balancing remifentanil and sevoflurane. The patient presented a decreased saturation (82%) and hypotension (mean arterial pressure [MAP] <45mmHg) with bradycardia (60 bpm), during the canulation of the ophthalmic artery. Epinephrine 100ucg was administered and improved the hemodynamic and respiratory parameters. The procedure was finalized without any additional complications and the patient was extubated after the procedure. 

## Discussion

Japanese investigators were the first to administer intravitreal and intraarterial chemotherapy for advanced or recurrent retinoblastoma [4]. From their experience, SOAC has been considered a safe procedure, with few complications mainly associated with vitreous hemorrhage, atrophia of the pigmented tissue of the retina, retinal detachment, and microemboli in the eye. Some publications have reported respiratory alterations associated with the canulation of the ophthalmic artery. Phillis et al. described a case series that reported an incidence of respiratory alterations in 24% of cases [5], and Kato et al., in a retrospective study, reported in 64% of patients a severe decrease of pulmonary distension [6]. These changes are clinically similar to that of bronchospasm, which are associated with sudden changes in tidal volume and hypoxemia, leading to hypotension and bradycardia. 

The cardiorespiratory events that occur due to the SOAC have been described previously [5-7], and they occur in 20-30% of patients [6]. The symptoms are similar to bronchospasm or anaphylaxis. The onset of these symptoms correlates with the catheterization of the ophthalmic artery and primarily affects pulmonary distensibility [7].

Although the mechanism of this physiological reaction has not been elucidated yet, some attribute this respiratory reaction to autonomic reflexes not described yet. Both the ophthalmic artery and the dura that surround it are innervated by the sensory branches of the trigeminal nerve [8], it is likely that there is some relationship between the intracranial vasculature and the airways. This cardiorespiratory response is related to vasovagal reflexes similar to the trigeminal-cardiac and oculo-respiratory reflex. The trigeminal cardiac reflex can cause a wide variety of arrhythmias and hypotension. The cardiovascular changes are mediated by an increase of the sympathetic activity from the vagal nerve and managed with anticholinergic drugs. The oculo-respiratory reflex has been associated with a decrease in the respiratory rate and the tidal volume, leading to apnea and respiratory arrest [9,10].

The trigger of both the hemodynamic and respiratory changes is the cannulation of the ophthalmic artery, even without administration of any substance through the catheter [5]. When these changes occur, the provider must stop the manipulation of the catheter immediately. The hypotension can present from 13-37 minutes [11], while the bradycardia might persist despite the treatment with atropine [5]. No reports to date have documented the requirement of a pacemaker. 

The alterations of the respiratory function are manifested with the decrease of tidal volume or an abrupt increase of the inspiratory pressure. Three possible conditions could cause the previous findings, pulmonary edema, anaphylactic shock, or bronchospasm. Since there were no secretions through the orotracheal tube, and there was a quick resolution, the diagnosis of edema was unlikely. The negative levels of tryptase in a case series [12] decrease the likelihood accounting for these symptoms for an anaphylactic reaction. 

These complications ought to be treated promptly since they debut with severe respiratory compromise [5]. To date, there is no consensus regarding optimal management; however, our experience and prior authors agree in certain aspects. Oxygen support to 100% must be instated as soon as possible, and early use of epinephrine should be instated (1 ucg/Kg up to 10 ucg/Kg) [5,12] even if there isn't hemodynamic compromise since this has been shown to improve bronchospasm significantly [12].

## Conclusions

Although it is worth noting that both in our case and prior case reports, these severe cardiorespiratory adverse events occur during the second session of SOAC, there is no clear explanation for this phenomenon. With the rapid advent of the endovascular approach for the treatment of RB in the pediatric population, understanding and learning how to manage the possible cardiovascular complications associated with these procedures, even if they are rare as described in our two cases, is paramount to achieve a higher quality of care. As this practice spreads as the standard of treatment, more descriptions of these complications could help us understand them better.
